# Changes in Health Insurance Coverage Over Time by Immigration Status Among US Older Adults, 1992-2016

**DOI:** 10.1001/jamanetworkopen.2020.0731

**Published:** 2020-03-11

**Authors:** Jessica Cobian, Maynor G. González, Ying J. Cao, Huiwen Xu, Rui Li, Morgan Mendis, Katia Noyes, Adan Z. Becerra

**Affiliations:** 1 School of Public Affairs, American University, Washington, DC; 2 Independent Researcher, Los Angeles, California; 3 Department of Epidemiology and Environmental Health, The State University of New York at Buffalo, Buffalo; 4 Department of Surgery, University of Rochester Medical Center, Rochester, New York; 5 Department of Public Health Sciences, University of Rochester Medical Center, Rochester, New York; 6 Inspire, Arlington, Virginia; 7 Social & Scientific Systems, Silver Spring, Maryland

## Abstract

**Importance:**

Disparities in health insurance coverage by immigration status are well documented; however, there are few data comparing long-term changes in insurance coverage between immigrant and nonimmigrant adults as they age into older adulthood.

**Objective:**

To compare longitudinal changes in insurance coverage over 24 years of follow-up between recent immigrant, early immigrant, and nonimmigrant adults in the US.

**Design, Setting, and Participants:**

This population-based cohort study used data from the nationally representative Health and Retirement Study. Data were collected biennially from 1992 to 2016. The population included community-dwelling US adults born between 1931 and 1941 and aged 51 to 61 years at baseline. Statistical analysis was performed from February 3, 2017, to January 10, 2020.

**Exposures:**

Participants were categorized as nonimmigrants (born in the US), early immigrants (immigrated to the US before the age of 18 years), and recent immigrants (immigrated to the US from the age of 18 years onward).

**Main Outcomes and Measures:**

Self-reported data on public, employer, long-term care, and other private insurance were used to define any insurance coverage. Longitudinal changes in insurance coverage were examined over time by immigration status using generalized estimating equations accounting for inverse probability of attrition weights. The association between immigration status and continuous insurance coverage was also evaluated.

**Results:**

A total of 9691 participants were included (mean [SD] age, 56.0 [3.2] years; 5111 [52.6%] female). Nonimmigrants composed 90% (n = 8649) of the cohort; early immigrants, 2% (n = 201); and recent immigrants, 8% (n = 841). Insurance coverage increased from 68%, 83%, and 86% of recent immigrant, early immigrant, and nonimmigrant older adults, respectively, in 1992 to 97%, 100%, and 99% in 2016. After accounting for selective attrition, recent immigrants were 15% less likely than nonimmigrants to have any insurance at baseline (risk ratio, 0.85; 95% CI, 0.82-0.88), driven by lower rates of private insurance. However, disparities in insurance decreased incrementally over time and were eliminated, such that insurance coverage rates were similar between groups as participants attained Medicare age eligibility. Furthermore, recent immigrants were less likely than nonimmigrants to be continuously insured (risk ratio, 0.89; 95% CI, 0.85-0.94).

**Conclusions and Relevance:**

Among community-dwelling adults who were not age eligible for Medicare, recent immigrants had lower rates of health insurance, but this disparity was eliminated over the 24-year follow-up period because of uptake of public insurance among all participants. Future studies should evaluate policies and health care reforms aimed at reducing disparities among vulnerable populations such as recent immigrants who are not age eligible for Medicare.

## Key Points

**Question:**

Does health insurance coverage over time differ between recent immigrant, early immigrant, and nonimmigrant adults in the US?

**Findings:**

In this cohort study of 9691 US adults, recent immigrants were 15% less likely than nonimmigrants to have insurance coverage at baseline, but this disparity was eliminated over the 24-year follow-up period in association with large increases in public insurance.

**Meaning:**

These findings suggest that public insurance programs such as Medicare play a prominent role in reducing disparities in insurance coverage for recent immigrants compared with nonimmigrants.

## Teaser

This cohort study compares longitudinal changes in insurance coverage over 24 years of follow-up between recent immigrant, early immigrant, and nonimmigrant adults in the US.

## Introduction

In 2017, the number of immigrants living in the US was reported to be 43.3 million, accounting for 14% of the nation’s population, compared with 6% in 1970.^1^ Similarly, the number of older adults in the US increased from 37.2 million in 2006 to 49.2 million in 2016.^2^ These numbers are expected to increase^3,4^; therefore, monitoring health insurance coverage among older US immigrants has become a nationwide priority. Previous research^5,6,7^ has shown that although the magnitude of outcomes varies, uninsured older adults have lower access to medical services, receive poorer quality of care, and experience worse outcomes compared with insured older adults.

Older immigrants represent a vulnerable population^8,9^ and face serious challenges acquiring insurance, including lack of access to enrollment services,^10^ language barriers,^11^ discrimination,^12^ and regulatory or statutory eligibility criteria for public insurance.^13^ For example, recent immigrants are not eligible for Medicaid or the Children’s Health Insurance Program coverage until 5 years after obtaining legal citizenship, a long waiting period that imposes substantial difficulties in receiving appropriate care. Thus, it is not surprising that many studies comparing insurance coverage by immigration status have shown that immigrants are more likely than nonimmigrants to be uninsured.^10,14,15,16,17^

Specifically, one study^15^ examined data from the Hispanic Health and Nutrition Examination Survey and reported lower rates of insurance coverage among Mexican and Cuban immigrants. Another national study^16^ showed that 56% of uninsured older adults were born outside the US. To date, most studies have been cross-sectional and few have evaluated longitudinal changes in insurance over time. Furthermore, despite the importance of Medicare age eligibility among older adults, to our knowledge, no study has compared insurance coverage by immigration status from before until after reaching Medicare age eligibility. Such an evaluation is likely to quantify disparities across life periods and reveal ways of confronting long-term disparities in insurance coverage.

To address this gap, we compared changes in insurance coverage by immigration status in a nationally representative cohort of older US adults who were followed for 24 years from before until after they reached Medicare age eligibility. We measured participants’ insurance coverage across 13 biennial waves from 1992 to 2016. We hypothesized that immigrants would have lower rates of insurance over time compared with nonimmigrants.

## Methods

### Data Source and Study Population

For this cohort study, we used publicly available data from the Health and Retirement Study (HRS), a longitudinal panel study that biennially surveys a sample of older US adults.^18^ The HRS began in 1992 and uses a multistage probability design to generate a nationally representative sample of older adults. The initial response rate was 82%, and wave-to-wave retention rates have been reported to be greater than 90%.^19^ The HRS is conducted at the University of Michigan in Ann Arbor and has been approved by the University of Michigan Health Sciences Human Subjects Committee. The HRS obtained oral informed consent from all participants, and we used deidentified, publicly available data; therefore, the institutional review board at the University of Rochester exempted the present study from review. This study follows the Strengthening the Reporting of Observational Studies in Epidemiology (STROBE) reporting guidelines.

We analyzed the RAND HRS file version P.^20^ Among 9762 HRS participants born between 1931 and 1941 who were enrolled at baseline in 1992, when they were aged 51 to 61 years, we excluded 71 participants with missing data on all outcome variables at baseline, leaving us with a sample of 9691 participants.

### Measures

The independent variable was immigration status, a 3-category variable that we defined as has been done before in the HRS.^21^ Participants were asked whether they were born outside the US, and if so, in what year they first immigrated. We created the following 3 mutually exclusive categories: (1) nonimmigrants (born in the US), (2) early immigrants (born outside the US who immigrated at ages 0-17 years), and recent immigrants (born outside the US who immigrated at age ≥18 years).

Our primary analysis consisted of longitudinal analyses evaluating changes in insurance by immigration status. Each biennial wave included data on self-reported public insurance, employer-based insurance (participant or spouse), and other private insurance (long-term care or other private insurance) at the time of interview. The primary outcome was any insurance. We also conducted analyses for public insurance, employer-based insurance, and other private insurance as secondary outcomes. Additional analyses classified participants as being continuously insured throughout the entire follow-up period (or until death or loss to follow-up).

### Potential Confounders

We distinguished between static confounders (ie, those that do not change over time) and those that are time varying. Static confounders included age at baseline, sex, race/ethnicity, education, father’s number of years of education, and religion. Time-varying confounders included wave number (categorical), marital status, household income, self-rated health, employment status, and interview language.

### Statistical Analysis

All analyses adjusted for the complex survey design by incorporating clustering and stratification variables derived by the HRS. We compared distributions of confounders by immigration status at the baseline wave. Our analytical approach for the primary analysis included adjusting for differential attrition found in longitudinal studies, induced by selective mortality and loss to follow-up that can bias estimates of risk factors. Selective attrition is also a concern in studies evaluating outcomes over time by immigration status because immigrants have better survival than nonimmigrants.^22,23,24^ Because insurance status is associated with mortality and attrition, differences in insurance by immigration status will be overestimated if attrition is ignored. To illustrate this potential for bias, we first estimated the adjusted association between immigration status and mortality by using a logistic regression model.

We accounted for selective attrition in longitudinal analyses using inverse probability of attrition (IPA) weights.^25,26^ We estimated weights for each observation in longitudinal analyses. For each wave of follow-up, we estimated separate weights on the basis of the reciprocal of the wave-specific probability of being alive and not lost to follow-up. We first estimated the IPA weight for being alive using pooled logistic regression models.^27^ At each wave of follow-up, we estimated the probability of surviving until that wave conditional on remaining alive and not lost to follow-up at the previous wave, wave number, static confounders, and time-varying confounders at the previous wave. Subsequently, we estimated the IPA weight for remaining not lost to follow-up using similar methods. The final weight for each observation was the product of the IPA weight for survival, the IPA weight for not lost to follow-up, and the HRS sampling weight at baseline.

We proceeded with our primary analysis by specifying a series of IPA-weighted generalized estimating equations using a sandwich variance estimator with a log-link (also known as a modified Poisson regression) with an unstructured covariance matrix to account for multiple observations per participant. We chose an unstructured covariance matrix because in all instances, it minimized the quasi-likelihood information criteria.^28^ The models regressed insurance on static covariates, immigration status, wave number, and cross-product terms for immigration status and wave number. If the cross-product term was statistically significant using a 2-sided *P* value and α = .05, we estimated wave-specific risk ratios (RRs) comparing insurance by immigration status because the association was heterogeneous across waves. Otherwise, the cross-product term was removed from the model, and a single overall RR was calculated.

For secondary analyses, we used generalized-estimating equation models using a sandwich variance estimator with a log-link and an unstructured correlation matrix to estimate the adjusted association between immigration status and being continuously insured. Models were adjusted for baseline confounders only.

We used SAS statistical software version 9.4 (SAS Institute) to generate the analysis data set and fit generalized estimating equation models. We used the R statistical software version 3.5.3 (R Project for Statistical Computing) package *survey* to estimate complex survey-adjusted statistics. Statistical analysis was performed from February 3, 2017, to January 10, 2020.

## Results

Among 9691 participants (mean [SD] age, 56.0 [3.2] years; 5111 [52.6%] female), nonimmigrants composed 90% (n = 8649) of the cohort; early immigrants, 2% (n = 201); and recent immigrants, 8% (n = 841). Table 1 presents the distribution of potential confounders at baseline, overall, and by immigration status. Age at baseline (mean [SD], 56.0 [3.1] years for recent immigrants, 55.0 [3.1] for early immigrants, and 56.0 [3.2] for nonimmigrants) and marital status (620 [74.3%] of recent immigrants, 140 [73.1%] of early immigrants, and 6341 [74.0%] of nonimmigrants were married) were similar across the groups. Recent and early immigrants were more likely than nonimmigrants to be Hispanic (427 [45.7%] and 73 [30.9%] vs 394 [4.1%]), highly educated (college or higher, 139 [18.0%] and 44 [23.9%] vs 1429 [17.0%]), have a father with a high level of education (≥13 years, 99 [12.4%] and 43 [23.0%] vs 844 [9.9%]), earn the highest quartile of household income (197 [26.7%] and 55 [32.9%] vs 2168 [27.4%]), self-report having excellent health (190 [24.1%] and 58 [30.9%] vs 1856 [22.0%]), be unemployed (53 [6.4%] and 9 [4.4%] vs 255 [3.0%]), identify as Catholic (517 (60.3%] and 91 [45.2%] vs 2027 [24.6%]), and to request that the interview be conducted in Spanish (331 [34.0%] and 33 [13.7%] vs 50 [0.4%]). Of the original 9691 participants, 3775 (39%) died and 1917 (20%) were lost to follow-up or did not respond to the survey by 2016.

**Table 1.** Characteristics of the Population at Wave 1 (1992), Overall and Stratified by Immigration Status

Characteristic	Participants, Unweighted No. (Weighted %)			
	Overall (Unweighted N = 9691 [100%])	Recent Immigrants (Unweighted n = 841 [8%])	Early Immigrants (Unweighted n = 201 [2%])	Nonimmigrants (n = 8649 [90%])
Female	5111 (52.6)	460 (54.4)	108 (51.6)	4543 (52.5)
Age, mean (SD), y	56.0 (3.2)	56.0 (3.1)	55.0 (3.2)	56.0 (3.2)
Race/ethnicity				
Non-Hispanic white	6941 (74.5)	241 (33.0)	99 (55.0)	6601 (78.8)
Hispanic	894 (8.1)	427 (45.7)	73 (30.9)	394 (4.1)
African American	1653 (15.3)	78 (9.1)	27 (13.1)	1548 (15.9)
Other	203 (2.1)	95 (12.2)	2 (1.1)	106 (1.2)
Education				
Less than high school	2574 (25.0)	408 (44.7)	68 (29.6)	2098 (23.1)
High school	3674 (38.5)	181 (22.8)	61 (29.7)	3432 (40.2)
Some college	1831 (19.2)	113 (14.6)	28 (16.9)	1690 (19.7)
College or higher	1612 (17.2)	139 (18.0)	44 (23.9)	1429 (17.0)
Father’s education, y				
Missing	1236 (12.2)	105 (11.4)	32 (13.3)	1099 (12.3)
0-6	2257 (21.9)	338 (37.9)	60 (25.7)	1859 (20.4)
7-8	2106 (22.5)	97 (12.8)	26 (14.5)	1983 (23.6)
9-12	3106 (33.0)	202 (25.4)	40 (23.4)	2864 (33.9)
≥13	986 (10.4)	99 (12.4)	43 (23.0)	844 (9.9)
Marital status				
Married	7101 (74.0)	620 (74.3)	140 (73.1)	6341 (74.0)
Divorced	1495 (15.0)	127 (15.2)	32 (13.6)	1336 (15.0)
Widow	637 (4.7)	46 (5.2)	17 (8.2)	574 (6.4)
Unmarried	458 (6.3)	48 (5.3)	12 (5.0)	398 (4.7)
Annual household income^a^				
Quartile 1	2443 (22.2)	237 (24.9)	46 (17.5)	2160 (22.1)
Quartile 2	2421 (24.7)	214 (24.5)	57 (27.9)	2150 (24.6)
Quartile 3	2407 (25.7)	193 (23.7)	43 (21.7)	2171 (25.9)
Quartile 4	2420 (27.4)	197 (26.7)	55 (32.9)	2168 (27.4)
Self-rated health				
Excellent	2104 (22.3)	190 (24.1)	58 (30.9)	1856 (22.0)
Very good	2708 (28.6)	149 (18.8)	47 (26.3)	2512 (29.6)
Good	2689 (27.3)	260 (30.5)	44 (20.1)	2385 (27.2)
Fair	1392 (13.9)	163 (18.2)	36 (14.7)	1193 (13.5)
Poor	798 (7.8)	79 (2.3)	16 (3.1)	703 (0.6)
Employment				
Works full time	5349 (55.2)	431 (51.7)	108 (54.0)	4810 (55.5)
Works part time	982 (10.4)	94 (11.5)	15 (8.6)	873 (10.4)
Retired	1598 (16.4)	76 (9.3)	31 (15.6)	1491 (17.1)
Disabled	412 (4.0)	40 (4.2)	7 (3.2)	365 (4.0)
Not in labor force	1033 (10.7)	147 (16.8)	31 (14.2)	855 (10.1)
Unemployed	317 (3.3)	53 (6.4)	9 (4.4)	255 (3.0)
Religion				
Protestant	6333 (64.1)	238 (28.6)	89 (44.0)	6006 (67.8)
Catholic	2635 (28.0)	517 (60.3)	91 (45.2)	2027 (24.6)
Jewish	157 (1.8)	12 (1.7)	6 (3.9)	139 (1.8)
None	467 (5.1)	30 (3.8)	11 (5.0)	426 (5.2)
Other	99 (9.1)	44 (5.6)	4 (1.9)	51 (0.6)
Interview language				
English	9277 (96.6)	510 (66.0)	168 (86.3)	8599 (99.6)
Spanish	414 (3.4)	331 (34.0)	33 (13.7)	50 (0.4)

^a^ Annual household income quartiles are defined as follows: quartile 1, $0 to $18 000; quartile 2, $18 001 to $36 000; quartile 3, $36 001 to $59 800; and quartile 4, $59 801 or more.

The Figure depicts the percentage of participants who were insured at each wave, by immigration status. In 1992, 68%, 83%, and 86% of recent immigrant, early immigrant, and nonimmigrant older adults, respectively, reported having insurance coverage. At the end of follow-up in 2016, 97%, 100%, and 99% of recent immigrant, early immigrant, and nonimmigrant older adults, respectively, reported having insurance coverage. eFigure 1, eFigure 2, eFigure 3, and eFigure 4 in the Supplement show similar trends, but for individual types of insurance. At baseline, 5% of the sample had Medicare insurance, 1% had Medicaid insurance, and 1% had both Medicare and Medicaid coverage; these numbers were 91%, 7%, and less than 1%, respectively, by the end of follow-up. Employer-based insurance decreased over time for all immigration status groups. Other private types of insurance increased over time for all immigration status groups.

**Figure.**
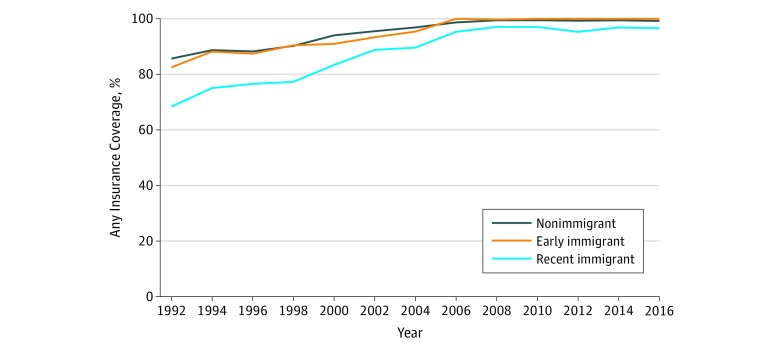
Any Insurance Coverage Over Time, by Immigration Status

In adjusted analyses comparing mortality by immigration status, both early (odds ratio, 0.79; 95% CI, 0.70-0.91) and recent (odds ratio, 0.84; 95% CI, 0.77-0.93) immigrants had lower odds of dying during the follow-up period compared with nonimmigrants, confirming differential attrition associated with selective mortality. Table 2 presents the main results from the primary analysis accounting for selective attrition for any insurance, which was the only insurance outcome that had statistically significant time trend effects (based on a global *F* test). The RRs and 95% CIs for immigration status associated with any insurance for each wave number are presented given that the time trend effect indicated statistically significant heterogeneity. The time trend beta coefficients on the log-risk scale for early and recent immigrants were 0.01 (95% CI, 0.002-0.021; *P* = .04) and 0.02 (95% CI, 0.018-0.022; *P* < .001), respectively, suggesting that both early and recent immigrants’ increases in any insurance over time were larger compared with nonimmigrants. This can be seen by the wave-specific RRs in Table 2. In 1992, recent immigrants were 15% less likely (RR, 0.85; 95% CI, 0.82-0.88) to have any insurance compared with nonimmigrants, but this discrepancy decreased over time and was eliminated by the end of follow-up in 2016 (RR, 1.02; 95% CI, 0.88-1.17). Similarly, early immigrants were 4% less likely (RR, 0.96; 95% CI, 0.91-1.01) to have any insurance compared with nonimmigrants, but this discrepancy decreased over time and was different by the end of follow-up (RR, 1.02; 95% CI, 0.81-1.23).

**Table 2.** Longitudinal Associations Between Immigration Status and Any Health Insurance Among Community-Dwelling Older Adults Accounting for Attrition, 1992-2016 (With Immigrant Time Trend Interaction)^a^

Immigration Status and Survey Year	Any Insurance, Risk Ratio (95% CI)
Nonimmigrant	1 [Reference]
Early immigrant	
1992	0.96 (0.91-1.01)
1994	0.98 (0.81-1.15)
1996	0.99 (0.81-1.17)
1998	1.01 (0.84-1.18)
2000	0.97 (0.80-1.14)
2002	1.02 (0.85-1.20)
2004	0.99 (0.80-1.16)
2006	1.01 (0.82-1.21)
2008	1.03 (0.83-1.22)
2010	1.05 (0.86-1.24)
2012	1.05 (0.85-1.26)
2014	1.05 (0.84-1.27)
2016	1.02 (0.81-1.23)
Recent immigrant	
1992	0.85 (0.82-0.88)
1994	0.88 (0.78-0.99)
1996	0.92 (0.78-1.06)
1998	0.89 (0.77-1.01)
2000	0.96 (0.84-1.08)
2002	1.02 (0.91-1.13)
2004	1.00 (0.88-1.12)
2006	1.04 (0.93-1.16)
2008	1.08 (0.94-1.23)
2010	1.03 (0.90-1.16)
2012	1.02 (0.87-1.18)
2014	1.05 (0.93-1.18)
2016	1.02 (0.88-1.17)

^a^ The model used a modified Poisson regression with a sandwich variance estimator, clustered by respondent, with an unstructured correlation matrix, and inverse probability of treatment weights for death and loss to follow-up.

Table 3 presents the main results from the primary analysis for public insurance, employer-based insurance, and other private insurance. Because the immigration status times wave number cross-product term was not statistically significant, there is only 1 effect estimate presented (no effect heterogeneity over time). The results suggested that recent immigrants were 16% less likely to have other private insurance (RR, 0.84; 95% CI, 0.73-0.96) compared with nonimmigrants regardless of the wave number (for public insurance, RR, 0.95 [95% CI, 0.91-1.00]; for employer-provided insurance, RR, 0.98 [95% CI, 0.84-1.12]). Furthermore, additional analysis suggested that recent immigrants were 11% less likely to be continuously insured (RR, 0.89; 95% CI, 0.85-0.94).

**Table 3.** Longitudinal Associations Between Immigration Status and Health Insurance Outcomes Among Community-Dwelling Older Adults Accounting for Attrition, 1992-2016 (No Immigrant Time Trend Interaction)^a^

Immigration Status	Insurance, Risk Ratio (95% CI)		
	Public	Employer	Other Private
Nonimmigrant	1 [Reference]	1 [Reference]	1 [Reference]
Early immigrant	1.05 (0.98-1.12)	0.95 (0.86-1.05)	1.14 (0.94-1.41)
Recent immigrant	0.95 (0.91-1.00)	0.98 (0.84-1.12)	0.84 (0.73-0.96)

^a^ The model used a modified Poisson regression with a sandwich variance estimator, clustered by respondent, with an unstructured correlation matrix, and inverse probability of attrition weights for death and loss to follow-up.

## Discussion

In this study of a nationally representative cohort of US adults who were not age eligible for Medicare at baseline, we followed immigrants and nonimmigrants from 1992 to 2016 and measured health insurance coverage biennially. We found disparities in any health insurance coverage between recent immigrants and nonimmigrants at baseline, predominantly associated with lower rates of private insurance plans among recent immigrants. However, these disparities in insurance coverage decreased incrementally over time such that the rates of any insurance coverage by immigration status did not differ as the cohort aged into older adulthood.

These trends were driven by public insurance coverage, which increased dramatically for all participants as the cohort became age eligible for Medicare. Furthermore, recent immigrants were less likely to be continuously insured. We did not find differences in rates of insurance coverage between early immigrants and nonimmigrants, which we hypothesize is associated with an increased awareness and knowledge about the US health care and insurance systems. These findings suggest that recent immigrants experience sociocultural and systemic barriers to attaining health insurance coverage, but these disparities can be ameliorated by access to public insurance programs such as Medicare. Accordingly, recent older adult immigrants are likely to rely on public insurance programs as a safety net to have equitable insurance coverage as compared with nonimmigrants.

The immigrant population in the US has been steadily increasing.^1^ Thus, ensuring that immigrants have equitable health insurance coverage remains a difficult challenge especially because US immigrants represent a heterogeneous group of individuals. Although immigrants face several barriers to obtaining high quality health care, numerous studies have shown that immigrants have superior health outcomes, including mortality. This observation has been termed the “healthy immigrant paradox” and has been the subject of investigation for decades.^22,23,24^ Although the reasons explaining this finding have yet to be definitely elucidated, it is likely a product of selection bias—that is, immigrants must meet certain health requirements to be allowed entry into the US^29^—and also by the fact that many immigrants come to the US for economically advantageous reasons.^30^ Thus, immigrants who come to the US are likely healthier than their counterparts born outside the US who do not immigrate to the US. In the present study, we observed that both recent immigrants and early immigrants were less likely to die during follow-up compared with nonimmigrants. Differential attrition associated with selective mortality over time may bias estimates when evaluating health insurance coverage by immigration status. To our knowledge, this is the first study to overcome this limitation by adjusting for selective attrition using IPA weights.

Our findings are consistent with prior cross-sectional studies that have generally reported lower rates of insurance among immigrants. Furthermore, our study corroborates 1 longitudinal study^31^ among adults showing that Mexican immigrants who immigrated early were more likely to have health care insurance compared with immigrants who immigrated recently. Another study^32^ assessed changes in insurance by immigration status among adults aged 70 years and older over a 6-year follow-up period and reported that recent immigrants were less likely to have Medicare insurance and private insurance. The mechanisms explaining why recent immigrants experience worse insurance coverage compared with their early immigrant counterparts have not been completely elucidated.^33,34^

Although previous studies have added valuable insight, the short follow-up periods of longitudinal studies and lack of adjustment for selective attrition have limited our understanding of changes in health insurance coverage by immigration status as they transition from middle age to older adulthood. Evaluating trends in insurance as participants age can lead to greater understanding of the mechanisms underlying disparities in insurance among recent immigrants and may highlight avenues for policy makers to equalize coverage rates for vulnerable populations. Our study addresses this crucial gap by following a nationally representative sample of US adults who were not Medicare age eligible at the start of the study and later gained age eligibility.

Our study contributes to the literature in 3 specific ways. First, it documents disparities in insurance between recent immigrants and nonimmigrants soon before Medicare age eligibility, driven by lower rates of private insurance. Contrary to a previous study, ours did not provide evidence showing that immigrants were less likely to have employer insurance. The previous study was conducted among a sample of adults of all ages, whereas the youngest adult at baseline in our study was aged 51 years. Given that employment and retirement dynamics are different as adults age, employment-based insurance may be less relevant for older adults. Second, it provides evidence suggesting that public insurance programs may alleviate disparities in insurance among vulnerable populations and, thus, may be a safety net for recent immigrants. Third, our analyses showing that recent immigrants were less likely to be continuously insured suggest that disparities in the extent of coverage also exist.

We believe these findings have timely ramifications for physicians and policy makers in the midst of an evolving health care system undergoing reform and increasingly demanding value. Given that immigrants are a sizable proportion of the overall population, the American College of Physicians^35,36^ has led several calls to action arguing that access to care for immigrants is a national public health issue. Although our study does not provide evidence of how best to proceed with health care reform, it suggests that future research should continue to investigate how to maximize the utility of public insurance programs. Furthermore, as advanced payment models increasingly develop community-level strategies for care coordination,^37^ individual physicians should keep in mind that these efforts might need to be emphasized among recent immigrants.

### Limitations

This study has several limitations that must be considered. First, insurance status was evaluated at 1 time point at each biennial wave. Second, we were not able to ascertain whether immigrants were undocumented. Estimating what percentage of uninsured immigrants were documented vs undocumented could identify reasons why immigrants were insured at a lower rate at baseline, because undocumented immigrants are not eligible for public insurance. Nevertheless, highlighting disparities among all immigrants is still important given that recent immigration policy efforts have been discussed to grant undocumented immigrants the same eligibility as documented immigrants. Third, our analyses assume that models have been specified correctly and that we have adequately adjusted for confounding. Fourth, our results are generalizable only to community-dwelling older adults born between 1931 and 1941. This may be important because older adult immigrants born in other periods may be different from immigrants who were born between 1931 and 1941. Future studies should examine whether these disparities are present across other cohorts of older adults. In addition, this study used the time period of arrival into the US as a baseline, but not the number of years living in the US because we were not able to ascertain whether immigrants moved back and forth between their native countries and the US. Accordingly, we were not able to measure the reasons why participants immigrated to the US, nor could we measure the patterns of insurance in earlier life periods.

## Conclusions

This study found disparities in insurance coverage between recent immigrant and nonimmigrant adults, although these disparities were eliminated over time as participants aged into Medicare eligibility. This study contributes to the ongoing conversation regarding eligibility criteria for documented and undocumented immigrants in accessing health care. Future studies should evaluate how to deliver value-based care to vulnerable populations such as older adult immigrants.^38,39,40^

## Notes

Supplement. **eFigure 1.** Medicare Insurance Coverage Over Time by Immigration Status **eFigure 2.** Medicaid Insurance Coverage Over Time by Immigration Status **eFigure 3. **Employer Insurance Coverage Over Time by Immigration Status **eFigure 4.** Other Insurance Coverage Over Time by Immigration Status
