# Supplementary material for: Changes in Health Insurance Coverage Over Time by Immigration Status Among US Older Adults, 1992-2016
Source: JAMA Netw Open. 2020 Mar 11;3(3):e200731. doi: 10.1001/jamanetworkopen.2020.0731 (PMC7066476; doi:10.1001/jamanetworkopen.2020.0731)
Supplement: Supplement. — eFigure 1. Medicare Insurance Coverage Over Time by Immigration Status eFigure 2. Medicaid Insurance Coverage Over Time by Immigration Status eFigure 3. Employer Insurance Coverage Over Time by Immigration Status eFigure 4. Other Insurance Coverage Over Time by Immigration Status [file jamanetwopen-3-e200731-s001.pdf]

Supplementary Online Content

Cobian J, González MG, Cao YJ, et al. Changes in health insurance coverage over time by immigration status among US older adults, 1992-2016. *JAMA Netw Open*. 2020;3(3):e200731. doi:10.1001/jamanetworkopen.2020.0731

eFigure 1. Medicare Insurance Coverage Over Time by Immigration Status

eFigure 2. Medicaid Insurance Coverage Over Time by Immigration Status

eFigure 3. Employer Insurance Coverage Over Time by Immigration Status

eFigure 4. Other Insurance Coverage Over Time by Immigration Status

This supplementary material has been provided by the authors to give readers additional information about their work.

© 2020 Cobian J et al. *JAMA Network Open.*

eFigure 1: Medicare Insurance Coverage Over Time by Immigration Status

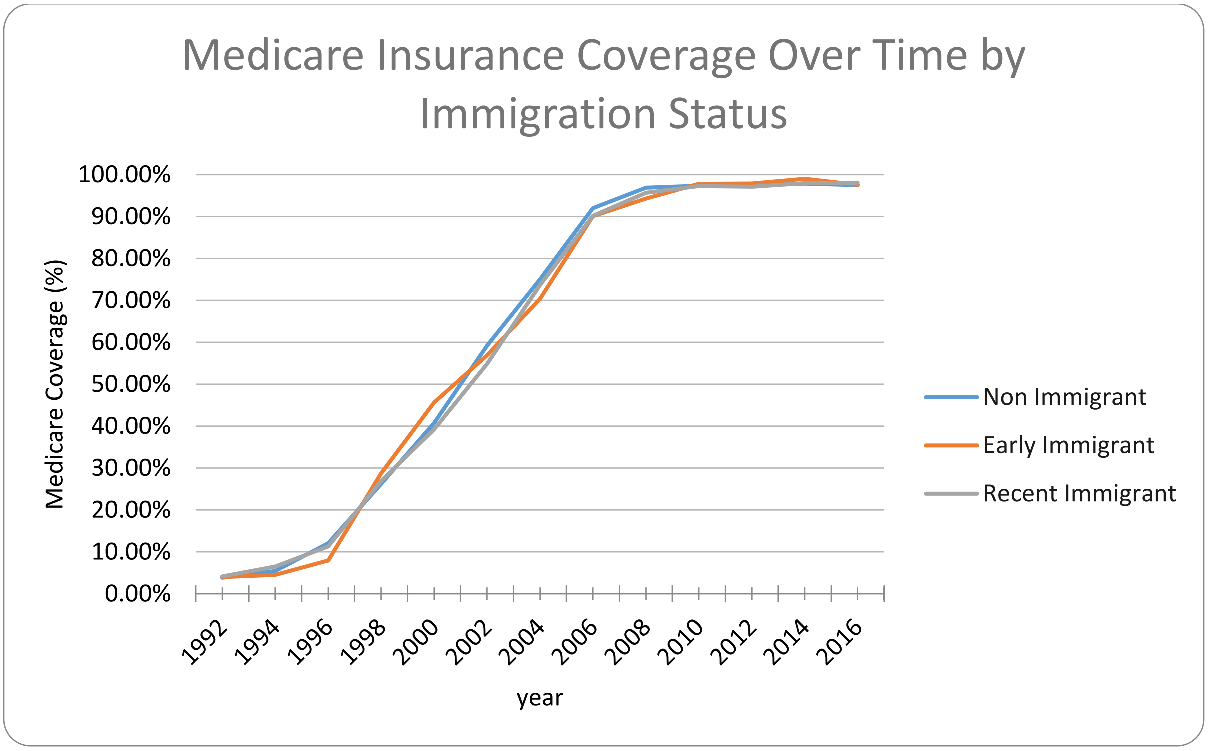

© 2020 Cobian J et al. *JAMA Network Open.*

eFigure 2: Medicaid Insurance Coverage Over Time by Immigration Status

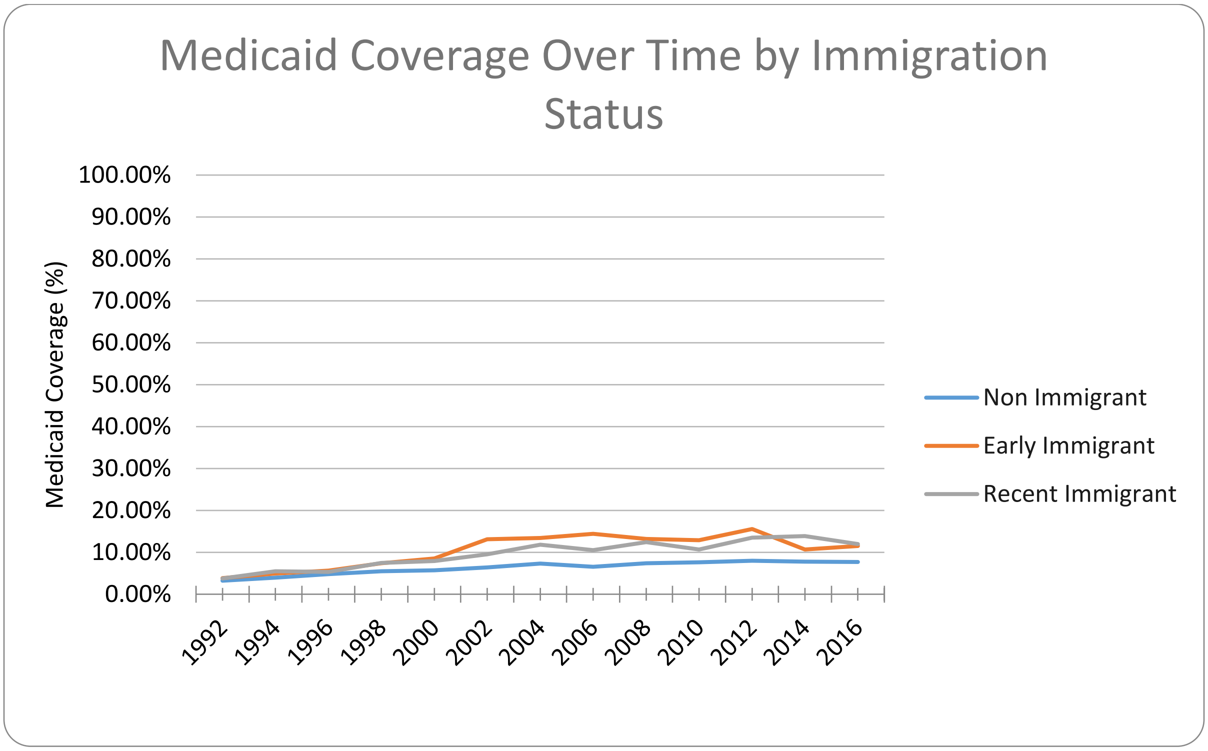

© 2020 Cobian J et al. *JAMA Network Open.*

eFigure 3: Employer Insurance Coverage Over Time by Immigration Status

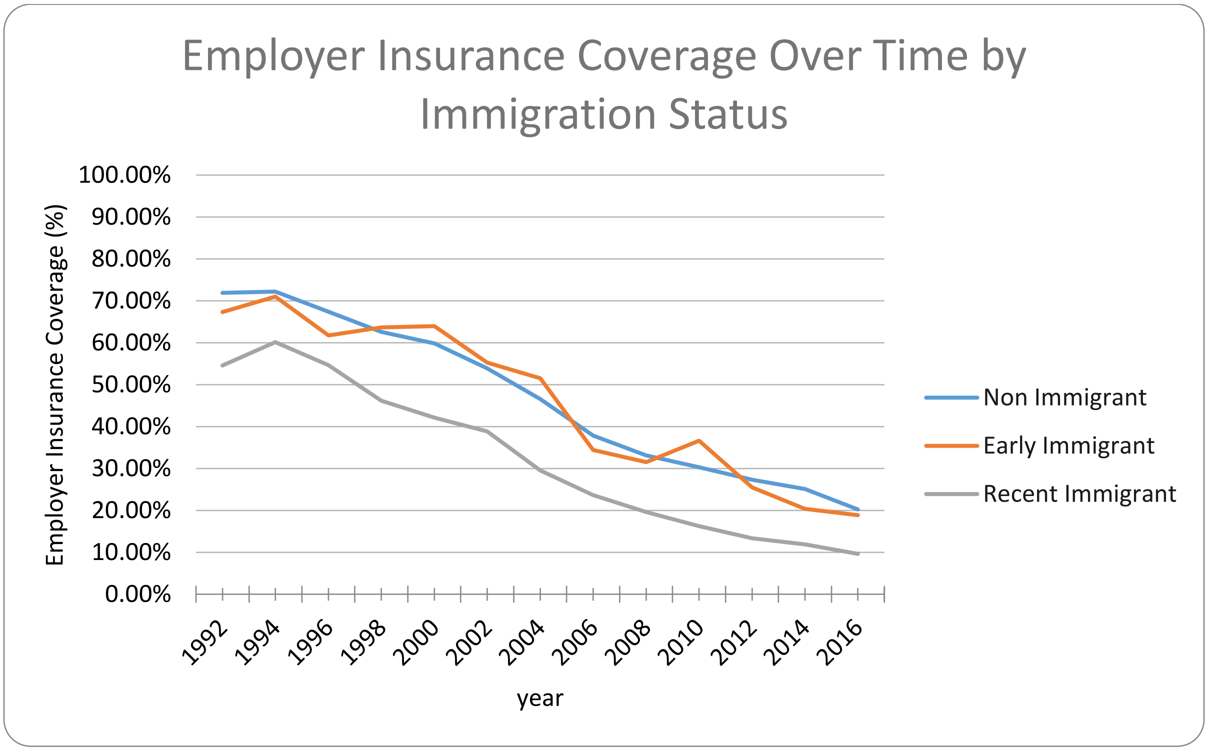

© 2020 Cobian J et al. *JAMA Network Open.*

eFigure 4: Other Insurance Coverage Over Time by Immigration Status

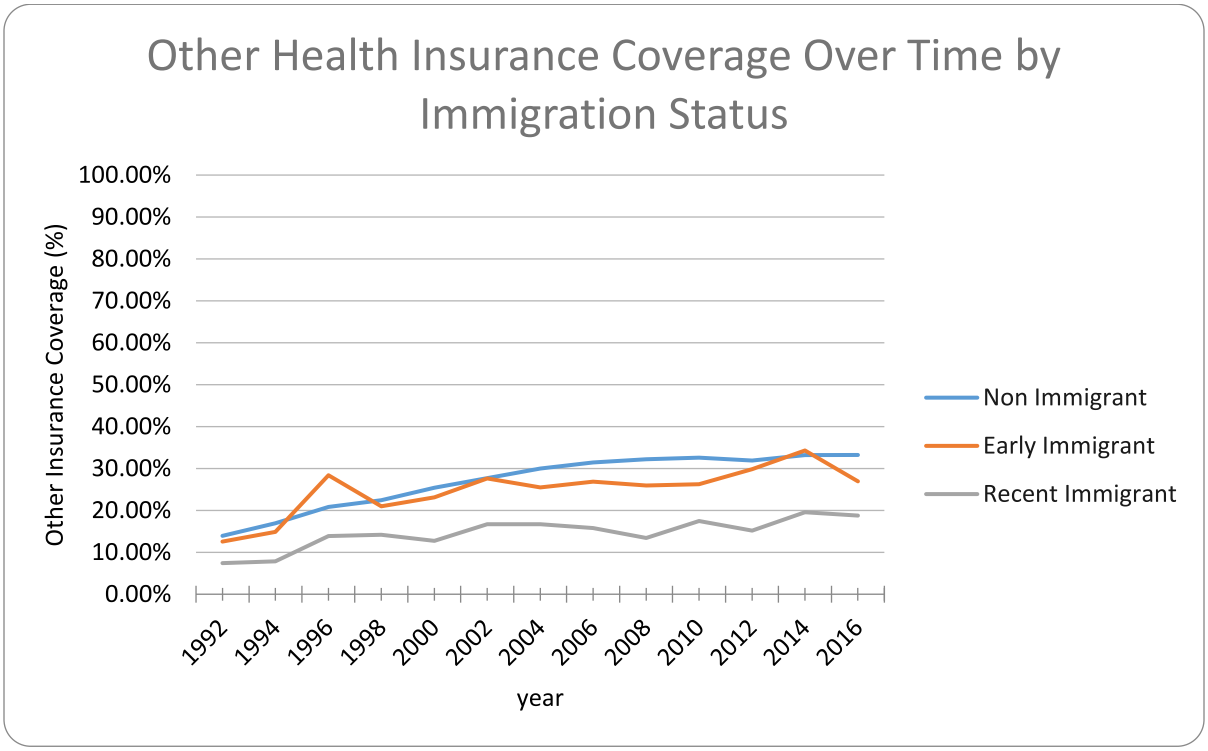

© 2020 Cobian J et al. *JAMA Network Open.*
